# Roadblocks and COVID-19: Hope and Strict Control

**DOI:** 10.1017/dmp.2020.208

**Published:** 2020-06-24

**Authors:** Beuy Joob, Viroj Wiwanitkit

**Affiliations:** Private Academic Practice, Bangkok, Thailand; Ajeenkya D Y Patil University, Pune, India; Hainan Medical University, Haikou, China

**Keywords:** COVID-19, disaster medicine, infection control

We read the publication titled “Roadblocks to Infection Prevention Efforts in Healthcare SARS-CoV-2/COVID-19 Response” with great interest.^1^ Popescu noted that “Awareness of these competing priorities and the challenges infection prevention programs face when working to maintain biopreparedness is critical in adequately addressing this critical infrastructure in the face of an international outbreak."^1^ Our setting, Thailand, was the second country with a documented COVID-19 patient case.^2^ The disease has existed for many months and several infection control attempts, including implementing a curfew, have been used. The priorities might be important, but strict control requires a broad collaboration. In Thailand, the local Center of Disease Control follows International Health Regulations^3-5^ with worldwide standard infection control measures followed. Public place closures, limitation of public transportation, and promotion of social distancing are observed.^4^ In fact, in many countries some people are still venturing outside with limited transportation, demonstrating that the social distancing campaign may be less than successful.^6^ Insufficient supplies of personal protective equipment and face masks are another serious problem.^7^ Similar to many countries, there is a requirement for valid risk management strategies in Thailand.^8^ Surprisingly, some local primary care center staff have poor knowledge of the disease and do not recognize the standard disease control concept.^9^

There must be hope for controlling COVID-19, but it must be based on a directional plan against the emerging disease.
